# GPTransformer: A Transformer-Based Deep Learning Method for Predicting Fusarium Related Traits in Barley

**DOI:** 10.3389/fpls.2021.761402

**Published:** 2021-12-16

**Authors:** Sheikh Jubair, James R. Tucker, Nathan Henderson, Colin W. Hiebert, Ana Badea, Michael Domaratzki, W. G. Dilantha Fernando

**Affiliations:** ^1^Department of Computer Science, University of Manitoba, Winnipeg, MB, Canada; ^2^Department of Plant Science, University of Manitoba, Winnipeg, MB, Canada; ^3^Brandon Research and Development Centre, Agriculture and Agri-Food Canada, Brandon, MB, Canada; ^4^Morden Research and Development Centre, Agriculture and Agri-Food Canada, Morden, MB, Canada; ^5^Department of Computer Science, University of Western Ontario, London, ON, Canada

**Keywords:** genomic prediction, deep learning, transformer, feature selection, quantitative traits, barley, fusarium head blight, deoxynivalenol

## Abstract

Fusarium head blight (FHB) incited by *Fusarium graminearum* Schwabe is a devastating disease of barley and other cereal crops worldwide. Fusarium head blight is associated with trichothecene mycotoxins such as deoxynivalenol (DON), which contaminates grains, making them unfit for malting or animal feed industries. While genetically resistant cultivars offer the best economic and environmentally responsible means to mitigate disease, parent lines with adequate resistance are limited in barley. Resistance breeding based upon quantitative genetic gains has been slow to date, due to intensive labor requirements of disease nurseries. The production of a high-throughput genome-wide molecular marker assembly for barley permits use in development of genomic prediction models for traits of economic importance to this crop. A diverse panel consisting of 400 two-row spring barley lines was assembled to focus on Canadian barley breeding programs. The panel was evaluated for FHB and DON content in three environments and over 2 years. Moreover, it was genotyped using an Illumina Infinium High-Throughput Screening (HTS) iSelect custom beadchip array of single nucleotide polymorphic molecular markers (50 K SNP), where over 23 K molecular markers were polymorphic. Genomic prediction has been demonstrated to successfully reduce FHB and DON content in cereals using various statistical models. Herein, we have studied an alternative method based on machine learning and compare it with a statistical approach. The bi-allelic SNPs represented pairs of alleles and were encoded in two ways: as categorical (–1, 0, 1) or using Hardy-Weinberg probability frequencies. This was followed by selecting essential genomic markers for phenotype prediction. Subsequently, a Transformer-based deep learning algorithm was applied to predict FHB and DON. Apart from the Transformer method, a Residual Fully Connected Neural Network (RFCNN) was also applied. Pearson correlation coefficients were calculated to compare true vs. predicted outputs. Models which included all markers generally showed marginal improvement in prediction. Hardy-Weinberg encoding generally improved correlation for FHB (6.9%) and DON (9.6%) for the Transformer network. This study suggests the potential of the Transformer based method as an alternative to the popular BLUP model for genomic prediction of complex traits such as FHB or DON, having performed equally or better than existing machine learning and statistical methods.

## 1. Introduction

Barley (*Hordeum vulgare* L.) is one of the most ancient grains, and is currently the fourth-most produced cereal globally measured both in area harvested and yield (FAO, 2019). Barley is primarily grown as animal fodder, or used by the malting and brewing industries. As a cash crop, malting barley necessitates maximized yield performance, and requires strict management of numerous grain-quality characteristics with specific parameter ranges (Izydorczyk and Edney, 2017). Barley achieving these superior standards can be sold into the lucrative malting barley market, where it returns a significant premium to the barley producer. Fusarium head blight (FHB), caused by *Fusarium graminearum* Schwabe [teleomorph: *Gibberella zeae* (Schwein.) Petch], is a devastating disease of barley. Primary concern of the disease is due to associated trichothecene mycotoxins such as deoxynivalenol (DON), which are potent inhibitors of protein synthesis (Pestka, 2010). Due to potential adverse toxic effects, DON along with its alternative forms are highly regulated with maximum consumption limits set for humans and livestock (EFSA CONTAM Panel, 2017).

Breeding FHB resistant varieties is a sustainable disease management solution, which has been achieved mainly through large disease nurseries. Studies have generally a demonstrated positive association between visual symptoms of FHB infection and DON content in matured grains. However, this correlation is often moderate at best (Buerstmayr et al., 2004; Choo et al., 2004; He et al., 2015; Huang et al., 2018; Tucker et al., 2019). Mycotoxin quantification is highly technical, where sampling protocols, quality controls and choice of analytical technologies are all implicated as important factors (Tittlemier et al., 2021). Analytical chemistries are expensive and labor requirements for harvest and processing grains are substantial.

FHB and DON content resistances are both under quantitative genetic control in barley (affected by many genes, each with a small effect). Significant undertakings have been made in genetic studies of biparental populations to identify quantitative trait loci (QTL) for breeding resistant barley cultivars (Fernando et al., 2021). While QTLs have been identified for FHB and DON, they are limited by the minimal level of genetic variance they explain, environmental specificity, and common association with negative agronomics such as extreme heading date and tall stature. Incorporating major QTLs from moderately resistant source “Chevron” such as Qrgz-2H-8 into elite backgrounds, did not result in sufficient resistance levels (Linkmeyer et al., 2013). Some commercial success has been achieved in developing moderately resistant cultivars such as six-row, malting barley “Quest,” through pyramiding of multiple resistances (Smith et al., 2013). Association mapping was able to identify QTLs independent of negative agronomic traits, however these were small, only explaining 1–3% of the observed variance (Massman et al., 2011).

Cereal crops (Poaceae family, Triticeae tribe) are characterized by their large genomes, with frequent repetitive elements (Mascher et al., 2017). With plummeting cost of genomic tools and availability of highly improved reference genomes, modern breeding approaches are now possible, which take advantage of genome-wide marker capabilities for the use in predicting complex traits (Jannink et al., 2010). In the face of this challenge, genomic prediction of FHB has been possible using statistically based methodologies in hexaploid (bread) wheat (Rutkoski et al., 2012; Arruda et al., 2015; Jiang et al., 2015; Mirdita et al., 2015; Hoffstetter et al., 2016; Dong et al., 2018); durum (pasta) wheat (Steiner et al., 2019; Moreno-Amores et al., 2020) and six-row barley (Sallam and Smith, 2016; Abed et al., 2018). While cereal genomes are complex, initial results of genomic prediction for FHB and DON content are very promising, and demand further investigation.

Traditional statistical algorithms such as Best Linear Unbiased Prediction (BLUP) and variants (Burgueño et al., 2012; Cuevas et al., 2017, 2019; Ferrão et al., 2017; Howard et al., 2019) have been applied in many genomic prediction problems. These models are mostly linear in nature and perform well for additive traits. Machine learning methods have been applied in genomic prediction with moderate success (Ogutu et al., 2011; Heslot et al., 2012; Poland et al., 2012; González-Camacho et al., 2018). The machine learning methods claim to capture non-additive effects better than the statistical methods (Heslot et al., 2012). Deep learning is a subset of machine learning that is gaining popularity for genomic prediction (Rachmatia et al., 2017; Ma et al., 2018; Jubair and Domaratzki, 2019; Khaki and Wang, 2019). Deep learning differs from traditional machine learning by applying multiple networks along with non-linear functions that often imitate how the human brain learns and identifies patterns based on the learned representations. Under the training phase of genomic prediction, these deep learning algorithms take inputs of genotype data of different varieties, and their corresponding phenotypes, to learn the parameters of the model. During the testing phase, only the genotype data of other varieties is used as input and the trained model predicts the corresponding phenotypes of the test data. These deep learning methods have performed equally or better than existing statistical methods (Ma et al., 2018; Jubair and Domaratzki, 2019; Khaki and Wang, 2019). For an overview of deep learning algorithms and their application in genomic prediction, we refer the readers to a recent review (Montesinos-López et al., 2021).

Neural networks such as feed-forward neural networks (Rachmatia et al., 2017; Khaki and Wang, 2019) and Convolutional Neural Networks (CNNs) (Ma et al., 2018; Jubair and Domaratzki, 2019) have been applied in genomic prediction. The feed-forward network can be compared to *n* linear regressions where these *n* linear regressions are the hidden neurons of the feed-forward network. The output neurons of the CNN also represents multiple linear regression models where the linear combination is produced from a very small subset of markers. CNN uses a sliding window allowing it to slide through the whole input space. Both feed-forward network and CNN do not reflect the polygenic interactive effects of markers as the relationship between markers are not considered in these algorithms.

Transformers are a family of deep learning algorithms that have been initially applied to Natural Language Processing (NLP) tasks such as classification, next sentence prediction and topic identification (Devlin et al., 2018; Radford et al., 2019; Raffel et al., 2019; Brown et al., 2020). Historically these methods perform well when trained on a large amount of data (Devlin et al., 2018; Radford et al., 2019; Raffel et al., 2019; Brown et al., 2020) and can be used for transfer learning. Apart from NLP, Transformer architecture has been successfully applied to other fields such as image processing (Dosovitskiy et al., 2020; Bazi et al., 2021). In this work, we proposed a Transformer-based genomic prediction model for predicting FHB and DON for barley. The Transformer consists of three main components: the self-attention, feed-forward networks, and layer normalization. The self-attention mechanism calculates the attention score for all genetic markers concerning a specific genetic marker (Vaswani et al., 2017) which helps to find the relation among markers. The layer normalization function converts each input marker to zero mean and unit variance. The Transformer network mainly identifies the inter-relation among markers.

Hardy-Weinberg equilibrium is a principle that states the allele frequency of a population will remain constant from generation to generation in the absence of disturbing factors (Acquaah, 2009). Under random mating, a population can obtain the equilibrium even after a single generation if there are no selection pressures. The principle also applies for the marker frequency and provides addition information about the population alongside genotype data (Acquaah, 2009). In this paper, we apply Hard-Weinberg equilibrium values as an input encoding for markers.

Hundreds of genes may contribute to a phenotype, such that identifying the top contributing genes and related markers is a challenging task. Feature selection algorithms identify essential features for a specific task (Saeys et al., 2007; Tang et al., 2014) and have been successfully applied in many classification problems of bioinformatics (Saeys et al., 2007). Mutual information is a filter based feature selection algorithm that identifies top features based on a set of classes and features. Top features identified using mutual information may represent targets which bear biological value.

In this work, we evaluate a Transformer-based deep learning method, GPTransformer, that uses genotypic and phenotypic data to predict FHB severity and DON levels in a two-row barley population. Our specific objectives were to (i) compare the accuracy of the GPTransformer model to existing genomic prediction methods, (ii) study the outcomes of the model if categorical encoding was used or marker frequency-based encoding was used, and (iii) investigate the effect of feature selection on genomic prediction using mutual information and examine the biological relevance of the top markers identified by the mutual information method. The Transformer network is trained using a graphical processing unit (GPU). As the internal mechanism of the Transformer creates a four dimensional matrix of size (batch size, number of heads, number of markers, number of markers) at certain point, which requires a large amount of GPU memory, the feature selection process also helps us to solve the GPU memory issue of the Transformer.

## 2. Methodology

### 2.1. Genotyping

The seed for a genetic panel was collected for a total of 400 spring habit two-row barley genotypes of mixed usage types of malt (171) and feed (229). Pure seed was provided by the Crop Development Center, University of Saskatchewan, Saskatoon, Canada (CDC) for a diversity panel of barley (92) breeding lines tested in the Western Canadian Cooperative Two-Row Barley Registration Trials (WCTBRT) 1994–2006 (Beattie et al., 2010). Additional elite lines (176) were selected from 2001 to 2013 WCTBRT based on past performance, with the majority from three breeding programs: CDC; Field Crop Development Center, Olds College; Agriculture and Agri-Food Canada (AAFC), Brandon Research and Development Center. Moreover, breeding lines (105) targeting FHB resistance and involving crosses to exotic sources, were also selected from these programs.

Two seeds were germinated on moist cotton balls for a week. At the two-leaf stage (Zadoks et al., 1974), leaves were cut from a single plant and flash-frozen in liquid nitrogen, then freeze-dried in a lyophilizer (Labconco Corporation, Kansas City, MO, USA). Genomic DNA was extracted from 100 mg of tissue using Qiagen, DNeasy 96 Plant Kit (Qiagen, Canada). The isolated DNA was evaluated by a NanodropTM 1000 spectrophotometer (Thermo Fisher Scientific Inc., Wilmington, DE, USA) for quality and concentration, then normalization to 50 ul mL^-1^. Samples were assayed on an Illumina iScan (Illumina, San Diego, CA, USA) using a custom iSelect-50 K SNP microarray (Bayer et al., 2017) at AAFC, Morden Research and Development Center, Morden, MB. A custom cluster file provided by M. Ganal (TraitGenetics GmbH, Germany) was used to call SNP alleles using Illumina GemomeStudio V2.0.5 software (Please see data availability statement). Data were filtered for ≥ 5% minor frequency alleles and ≤ 20% missing data.

### 2.2. Field Studies

FHB nurseries were grown in 2014 and 2015 at 3 locations: Brandon, Manitoba (49°51'56.0”N 99°58'57.7”W); Carman, Manitoba (49°29'52.9”N 98°02'19.2”W) and Carberry, Manitoba (49°54'16.6”N 99°21'19.0”W). The experiments followed a randomized complete block design at all locations (*n* = 2). Plots were sown with approximately 30–40 seeds and consisted of 0.9 m rows, 30 cm row spacing (Brandon, Carberry) or 1 m rows, 34 cm row spacing (Carman). Two inoculation methods were used. Brandon and Carberry experiments were inoculated by the grain spawn method, where maize kernels infected with 2 isolates each of 3ADON- and 15ADON-producing strains of *F. graminearum* were spread on the soil surface at 5 gm^−2^ at flag leaf then weekly for 3 total applications. Irrigation was applied after first inoculum application until all plots were rated. Experiments at Carman were sprayed with a macroconidia suspension of 3ADON and 15ADON isolates in equal proportions and standardized to 5 × 10^4^ spores ml^−1^. Plots were misted and sprayed at 75% spike emergence and then again 2 days following.

Plots were rated at the soft dough (Zadoks - Z85) stage. A visual scale (0–5) was used to evaluate a composite measure of incidence and severity (A. Tekauz, personal communication), where 0 = no infection. 1 = incidence low, up to 5% of spikes; severity low, 1 or 2 kernels per spike affected (up to 7% of head). 2 = incidence low to moderate, 5–15% of spikes infected; severity low to moderate, 1–4 kernels (up to 15% of head). 3 = incidence moderate, 15–30% of heads; severity moderate, 2–8 kernels (up to 25% of head). 4 = incidence moderate to high, 30–50% of spikes infected; severity moderate to high, 4–12 kernels (up to 40% of head). 5 = incidence high, 50% or more spikes affected; severity high, 5 to 15+ kernels (up to 50%+ of head diseased). Additional data were collected on days to heading (date 50% of row headed minus seeding date) at Brandon and Carberry and plant height (distance of soil surface to tip of spike excluding awns) at all locations.

Grains were harvested at maturity, using a stationary research combine with low wind speed setting, then dried in a high capacity drier for a few days. Grains were cleaned using an SLN3 sample cleaner (Pfeuffer GmbH, Kitzingen, Germany). A 20 g subsample was removed, cleaned free of debris and/or chaff then ground using a Perten 3610 lab mill with fine particle disc set (PertenElmer Inc. Waltham, MA, USA). Deoxynivalenol content was analyzed by enzyme-linked immunosorbent assay (ELISA) technique using Veratox^Ⓡ^ 5/5 (Neogen Corporation, Lansing, MI, USA) as per kit protocol (limit of detection = 0.1 mg kg^-1^). Samples were each tested in sub-sample pairs, where samples deviating by > 10% were repeated.

### 2.3. Allele Frequency Based Encoding

As the barley population is diploid, a locus *A* can have two alleles, *A* and *a* and three genotypes *AA*, *Aa*, and *aa*. We replaced the classical representation of genotype of a marker (1, 0, –1) with genotypic frequency by applying Hardy-Weinberg equilibrium (Acquaah, 2009). The frequency of alleles is calculated for each genetic marker. Suppose, the genotype *AA* and *Aa* appear *D* and *H* times respectively for a specific genetic marker. If the population size is *N*, the total number of alleles will be 2*N*. Then, the frequency of allele *A* for a specific genetic marker is calculated by applying Equation (1).

(1) $$\begin{array}{r} p=\frac{2D+H}{2N} \end{array}$$

The frequency of the allele *a* is *q*=1 − *p*. After calculating the allele frequency of a genetic marker, the expected genotypic frequencies of genotype *AA*, *Aa* and *aa* for a specific marker is obtained from *p*^2^, 2*pq*, and *q*^2^. These expected genotypic frequencies are used as marker values for the machine learning algorithms.

### 2.4. Transformer

**Figure 1:**
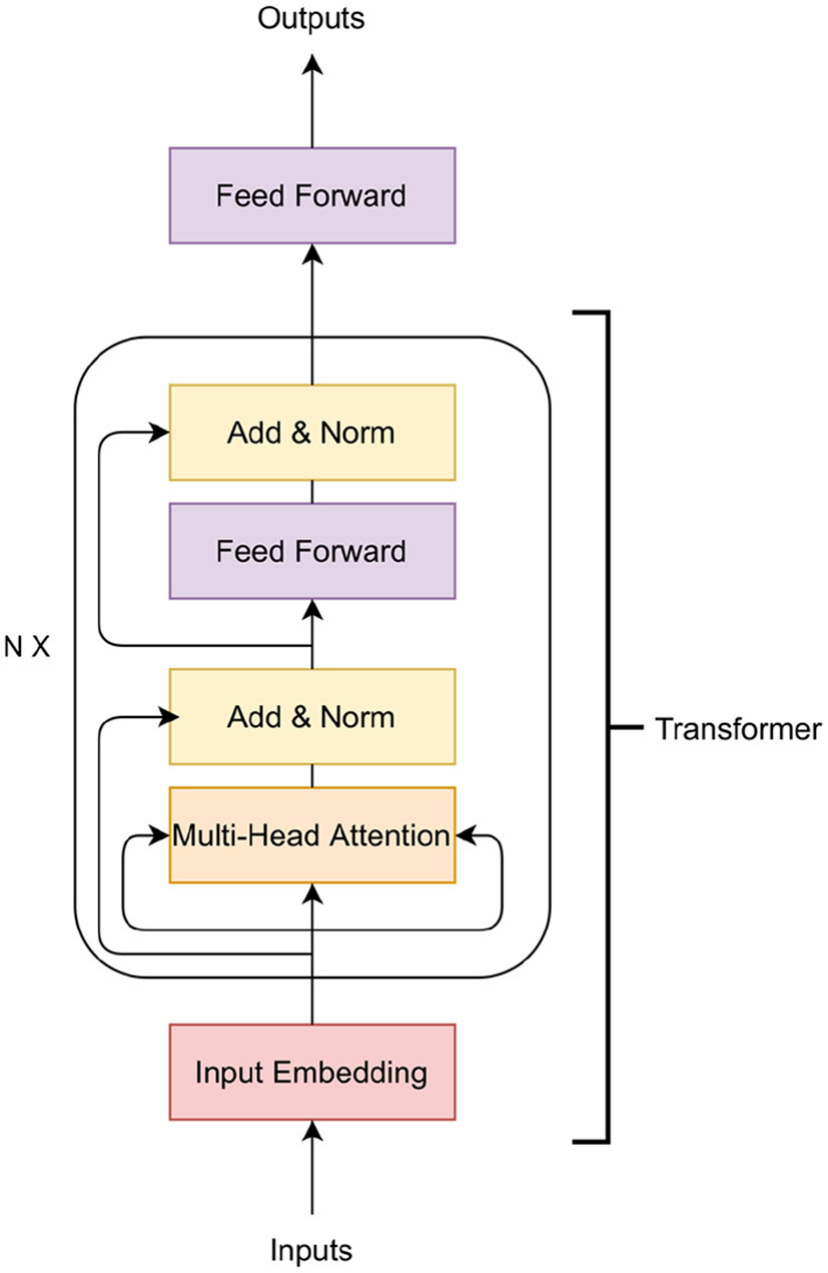
Transformer architecture.

In this paper, we examine the application of Transformer, a variety of neural networks. Deep neural networks have been applied to GS in the past. See the survey of Montesinos-López et al. (2021) for details on previous applications. The Transformer is a family of deep learning algorithms successfully applied to various NLP tasks such as classification, sequence to sequence modeling, and next sentence prediction. The Transformer architecture has two major components: i) encoder and ii) decoder (Vaswani et al., 2017). In this work, we use the encoder part of the Transformer along with an additional feed-forward network (Figure 1). The encoder of the Transformer architecture contains an embedding layer, a multi-head self-attention layer, and finally a feed-forward neural network.

The purpose of the embedding layer is to obtain an *n* = 8-dimensional expanded representation of markers. In this work, a feed-forward neural network is applied as the embedding layer. The input of the embedding layer is *m* markers and the output is an *m* × *n*-dimensional vector. The vector is then reshaped to an (*m, n*)-matrix to obtain *m* expanded representation of the markers. The output of the embedding layer is then passed to a multi-head attention network.

The multi-head attention network is based on the self-attention mechanism. The input of this layer is the expanded representation of the markers obtained in the embedding layer. The main building block of the multi-head-attention is the self-attention mechanism that calculates the attention score for all other expanded representation of markers with respect to a specific expanded representation. To calculate the self-attention, at first, each embedded marker creates three vectors: a query vector *q*, a key vector *k* and a value vector *v* by applying a linear transformation on the embedding. The query vector is the candidate expanded representation with respect to which the attention is measured while the keys are the set of expanded representations where the importance scores are assigned. The attention score is calculated as:

(2) $$\begin{array}{r} Attention\left(Q,K,V\right)=softmax\left(\frac{QK^{T}}{\sqrt{d_{k}}}\right)V \end{array}$$

In Equation (2), *Q* is a matrix of all the queries, *K* is a matrix of all the keys and *V* is a matrix of all the values and *d*_*k*_ is the dimension of keys. The last step is to generate a summation of the previous step, which produces the self-attention layer's output. In a multi-head attention setting, the Transformer model creates *h* independent linear representation from queries, keys and values. These *h* representations are then concatenated and passed through a linear projection layer to obtain the final output.

In Figure 1, a residual connection from the output of the input embedding layer is added to the output of multi-head attention. A layer-normalization is applied to the output of the residual connection. The Transformer block contains another feed-forward network and a layer-normalization after the feed-forward layer. The *N* in Figure 1 indicates that this Transformer block can be stacked *N* times and the output of the *Nth* encoder block will be the input of the feed-forward layer that predicts the phenotypes.

### 2.5. Residual Fully Connected Neural Network

**Figure 2:**
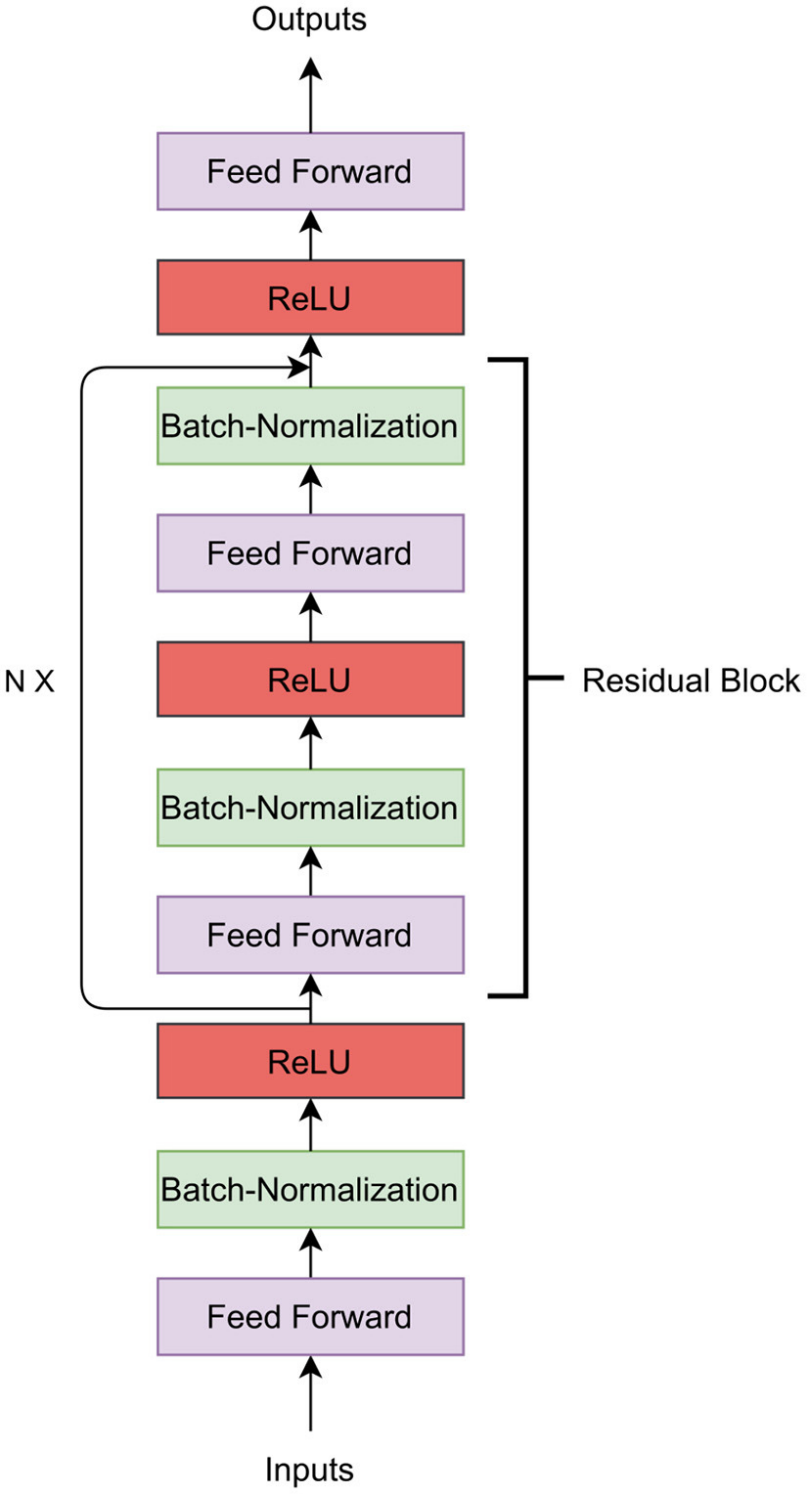
Residual fully connected network architecture.

We now describe our second deep learning model which is based on feed-forward network with residual connection. In general terms, a residual neural network is a neural network that has one or more residual connections. Residual connections allow skipping of layers in a neural network. In our implementation, the first layer of the Residual Fully Connected Neural Network (RFCNN) is a feed-forward neural network that takes *M* markers as the input and performs a linear transformation to produce an *n*-dimensional hidden representation of the markers. This hidden representation is the input of the batch-normalization layer. The batch-normalization layer normalizes the current batch by its mean and standard deviation. As the feed-forward network and batch normalization performs a linear operation on the input data, we apply the activation function ReLU to the batch-normalization layer's output. ReLU will return 0 if the input is ≤ 0; otherwise, it will return *x* where *x* is the input and thus, introduces the non-linearity. The output of ReLU is going to be the input of the next feed-forward network. In Figure 2, the residual connection is shown as the arrow on the left-side of the figure skipping over the intermediary layers. A residual connection is added from the output of each odd ReLU layer to each odd batch-normalization layer (except the first batch-normalization). The residual block can be stacked *N* times. The output layer is the feed-forward network that predicts the phenotypes.

### 2.6. Other Statistical and Machine Learning Models for Baseline Comparison

#### 2.6.1. Decision Tree

Decision Trees (DTs) are common statistical and machine learning methods used for predictive modeling. The baseline DT regressor models used in this analysis were built with the Scikit-learn (Pedregosa et al., 2011). Python module under default settings. To train the models, an input dataset of an *n* × *p* matrix of encoded genotypes and a *n*-dimensional vector of known corresponding phenotype responses were supplied. At each node, splits based on the SNP markers are considered and the highest quality split is chosen; in this case, the quality measure of the split is the mean squared error. This process continues recursively, splitting the data into subsets of instances at each internal node, until the branches terminate with leaf node and produce a response value. The values at the leaf nodes are the arithmetic mean of the known associated response variables to the instances that are present in the adjoining edge. With a fitted model, genotypes with unknown phenotype response can be predicted by iteratively testing the SNP marker values against the trees decision procedure until a leaf node is reached.

#### 2.6.2. Linear Regression

Like the DT models, the linear regression (LR) models used in this analysis were built with the Scikit learn Python module using default settings. LR fits a linear model by calculating coefficients on the independent terms that minimize the sum squared error between the known observations responses and the approximated predictions. The following is the form of the linear models:

(3) $$\begin{array}{r} \text{y}=\text{X}\beta +\varepsilon \end{array}$$

where **y** is the *n*-dimensional vector containing response variables (phenotype) for each of *n* input genotypes. **X** is the *n* × *p* matrix (*n* genotypes and *p* markers in each genotype), ***β*** is a *p*-dimensional column vector of unknown coefficient parameters, and ***ε*** is the *n*-dimensional unknown random error column vector. The linear regression model tries to learn ***β*** to make phenotype prediction.

#### 2.6.3. Ridge Regression Best Linear Unbiased Prediction

The ridge regression (RR) models were built using JMP Genomics 9 (JMP Genomics, 1989-2021). This process computes Best Linear Unbiased Predictions (BLUPs) that linearly correlate the genotypes, based on the input marker encodings, to a trait variable of interest. RR-BLUPs are linear mixed-models of the following from:

(4) $$\begin{array}{r} \text{y}=\text{F}\delta +\text{Z}\gamma +\varepsilon \end{array}$$

where **y** is the *n*-dimensional column vector containing response variables (phenotype) for each of *n* genotypes, **F** is the *n* × *q* matrix of known fixed-effects, ***δ*** is a *q*-dimensional column vector of unknown fixed-effects parameters, **Z** is the *n* × *p* matrix of known random-effects (*n* encoded genotypes), ***γ*** is the *p*-dimensional column vector of unknown random-effects parameters, finally, ***ε*** is the *n*-dimensional unknown random error column vector. It is assumed that the residuals ***ε*** and random-effects ***γ*** are normally distributed, $\varepsilon \sim N\left(0,\text{I}\sigma_{\varepsilon }^{2}\right)$ and $\gamma_{i}\sim N\left(0,\sigma_{\gamma_{i}}^{2}\right)$ where **I** is the identity matrix and $\sigma_{\gamma_{i}}^{2}$ are assumed equal for all SNP markers. The unknown model parameters are estimated from the solution of the mixed-model equations (Henderson, 1984). A scoring file is produced that contains an equation of a linear combination of SNP markers for predicting phenotype response from the testing set data.

### 2.7. Train-Test-Validation Split

To divide the data into train, test and validation sets, we follow the recommendation of Runcie and Cheng (2019). We randomly split the data in 85–15%, where 85% is the training data and the remaining 15% is the test data. From the training data, we again perform three random splits of 85–15%. The first 85% is the training set and the rest 15% is the validation set. Thus, from the data, we created three training sets, three validation sets and one test set.

To further investigate the reliability of the proposed GPTransformer model, we again perform *k* random splits of the dataset into 70–15–15% train-test-validation sets (*k* = 3). This time, in each split, the test set also changes along with the training and validation set.

### 2.8. Feature Selection

Feature selection methods identify features that contribute to a specific expression. In our work, the purpose of the feature selection is to identify those genetic markers that contribute toward low FHB or low DON levels. We applied mutual information feature selection, a filter based method, where the input is the genetic markers and the phenotypes and the output is a mutual information score (ranging 0-1). Discretization was performed where we divide the genotypes into three bins based on phenotypes (lowest 25%, middle 50%, highest 25%). The categories are the labels and the genetic markers are the features of mutual information algorithm which produces a mutual information score for each marker. The final mutual information score for each marker is the average mutual information score over the three training sets. Markers with average mutual information of ≥ 0.02 were selected.

### 2.9. Training Transformer

The input embedding of the Transformer network converts each marker to a eight-dimensional vector (hidden dimension, *n* = 8). Thus, if each genotype contains *m* genetic markers (*m* = 25, 000 before performing feature selection), the input embedding layer's output will be an (*m, n*) matrix. This (*m, n*) matrix is the input of the multi-head attention. As the multi-head attention computes pairwise attention between each marker, the operation will result in an (*h, m, m, n*) matrix where *h* is the number of heads. This operation has significant memory requirements for the GPU. For instance, on this dataset, it requires over 48 GB of memory.

To circumvent memory limitations, only selected features of mutual information are taken as the Transformer network input. We also pass only one genotype at each batch for training. Thus, the input of the embedding layer is all the markers of a genotype selected by the mutual information algorithm. The output is an (*f, n*) dimensional matrix where *f* is the number of markers. This (*f, n*) matrix will be the input of the Transformer encoder. Our Transformer neural network contains two Transformer encoder blocks (*N* = 2). We use two heads (*h* = 2) for each multi-head attention layer and each feed-forward block inside the Transformer encoder contains 256 hidden neurons. The final Transformer encoder's output is also an (*f, n*) matrix which is flattened to create a vector that contains *f* × *n* elements. This vector is the input of the last feed-forward network which contains one output neuron. We use the mean square error (MSE) loss function along with the Adam optimizer. The learning rate of the optimizer is 1*e* − 5. If there is no improvement in MSE loss in the validation set for ten consecutive epochs, we stopped the training.

### 2.10. Training Residual Fully Connected Neural Network

The first feed-forward layer takes *m* markers (all the markers) or *f* markers (feature selected) as an input and produces a 512-dimensional hidden representation. Each subsequent feed-forward layer takes the previous layer's input and produces a 512-dimensional hidden representation. The last feed-forward layer contains only one output neuron. We stack five residual blocks (*N* = 5) one after another. We also use MSE as the loss function and Adam as the optimizer with a learning rate of 1*e* − 5.

## 3. Results

The Pearson Correlation Coefficient (PCC) was calculated to measure the performance. The PCC calculates the linear relation between the true output and predicted output. The PCC value ranges from −1 to 1 where 1 indicates a perfect linear relation between the predicted phenotypes and the true phenotypes whereas −1 indicates the opposite relationship between the true and predicted phenotypes.

In the remainder of the paper, especially in the figure, we will denote genotype based encoding as HW, categorical encoding as CAT, Decision Tree algorithm as DT, Linear Regression as LR and Residual Fully Connected Neural Network as RFCNN.

### 3.1. Phenotype Assessment

**Figure 3:**
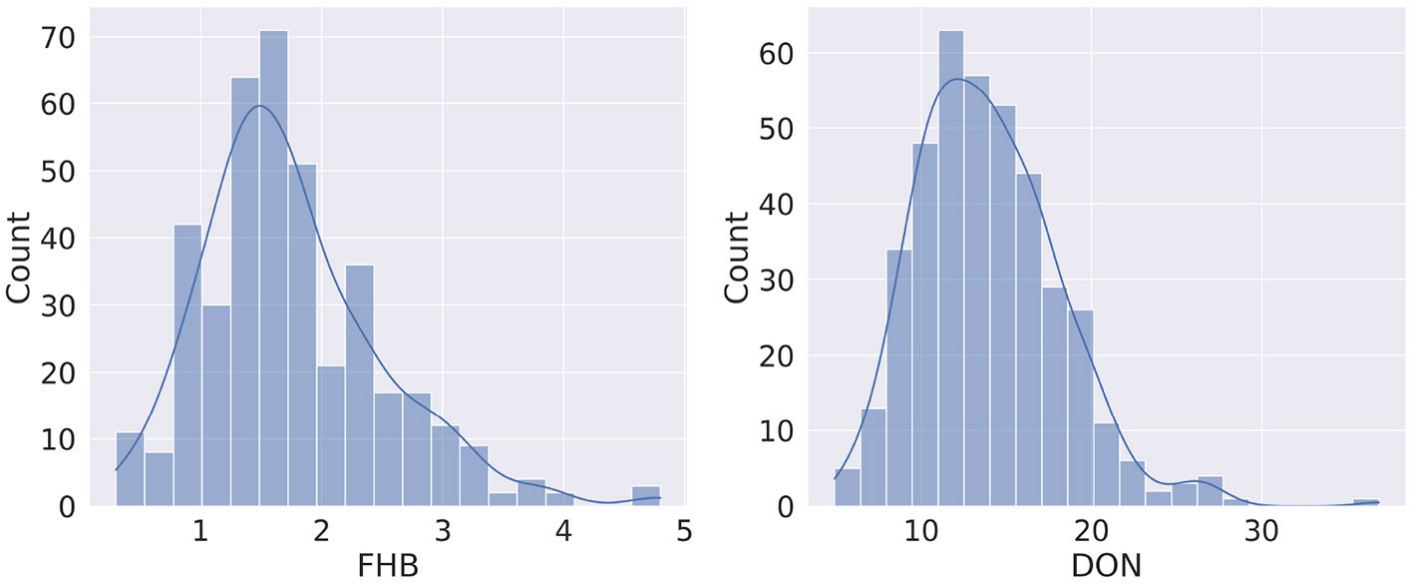
Distribution of phenotypes for Fusarium head blight (FHB, 0–5) and deoxynivalenol content (DON, *mg kg*^−1^).

The distribution of FHB and DON is shown in Figure 3. It is observed that the FHB values are distributed over 0.3–4.8 range (1.75 ± 0.04), while the DON values range from 4.9 to 36.9 mg kg^-1^ (13.96 ± 0.21) (Supplementary Table 1). For both phenotypes, the distribution curve is similar to normal distribution, however, a degree of positive skewness was observed in FHB (0.881) and DON (0.976). Shapiro-Wilk W tests conducted on FHB (*W* = 0.947, Prob <0.0001) and DON (*W* = 0.963, Prob <0.0001) indicated a degree departure from normality. Departures from normality were most obvious in tail regions of the distributions.

**Figure 4:**
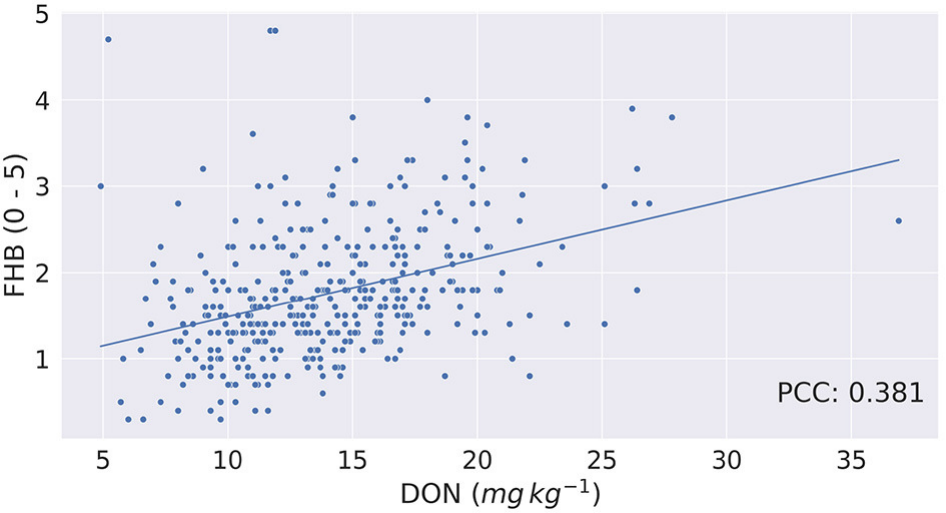
Fusarium head blight (FHB, 0–5) vs. deoxynivalenol content (DON, *mg kg*^−1^) for each of the barley genotype tested. Correlation between FHB and DON is 0.381.

Though from Figure 3 it seems there may be a linear relation between FHB and DON, it is found that the Pearson Correlation Coefficient (PCC) between the two phenotypes is 0.381 (*p* < 0.0001). Figure 4 shows a scatterplot of FHB vs. DON for each genotype. From Figure 4, it is observed that there is a very little correlation between the two phenotypes, which is reflected by the PCC score. This leads us to expect that similar genomic selection models may not immediately perform similarly when predicting the two phenotypes. FHB and DON were also examined for relationships with days to heading (*r* = −0.18, *P* < 0.004;0.18, *P* < 0.0003) and height (*r* = −0.60, *P* < 0.0001;−0.21, *P* < 0.0001).

### 3.2. Effect of Encoding Technique

**Figure 5:**
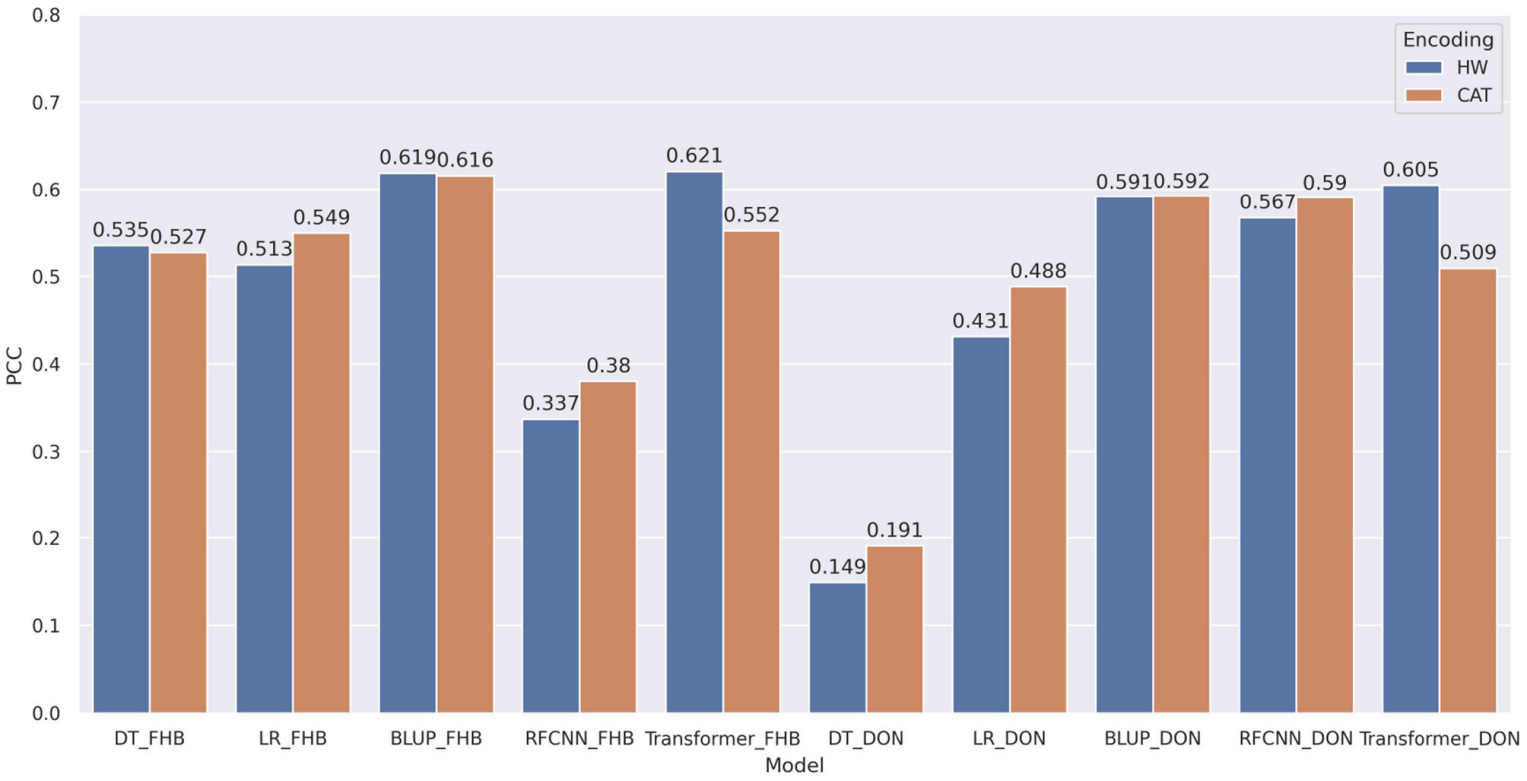
Comparison of pearson correlation coefficient based on encoding techniques. HW and CAT represents Hardy-Weinberg and categorical encoding. The correlation is measured between the target and predicted phenotypes. decision tree, linear regression, BLUP, residual fully connected neural network and transformer are applied for each encoding technique.

**Figure 6:**
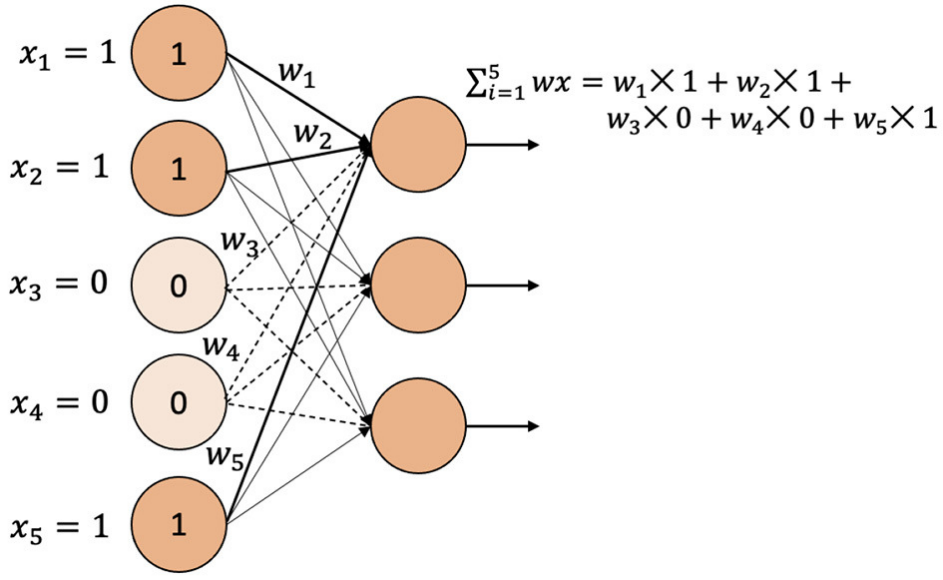
Categorical encoding when applied to a fully connected layer.

Two encoding techniques were implemented: i) categorical encoding (-1, 0, 1) and ii) genotype frequency-based encoding that follows Hardy-Weinberg equilibrium. Figure 5 shows the comparison of PCC between two encoding schemes for various models. In most of the models, the categorical encoding outperforms the genotype frequency-based encoding. In the BLUP, correlation is very close to each other for both traits as the correlation score varies from 0.001 to 0.003 based on different traits. For Transformer, Hardy-Weinberg encoding improves the correlation by 6.9% for FHB and 9.6% for DON. This can be explained by noting that, with the categorical encoding, the values of genotypes are 1, 0, and –1 and the heterozygous alleles will be considered neurons that do not have any effects. In particular, Figure 6 shows the effect of categorical encoding in the embedding layer. As the embedding layer's output is the input of the multi-head attention, multi-head attention also ignores any effect of heterozygous alleles. When applying genotype frequency-based encoding, different alleles of a specific gene have different values and these values even differ from gene to gene. For example, allele *AA* for gene *X* and allele *AA* for gene *Y* may appear in different frequencies and will have different Hardy-Weinberg values. Thus, the embedding layer does not suffer from multiplying-by-zero problems and improves the performance of the Transformer.

### 3.3. Effect of Feature Selection

No single molecular marker dominantly explained significant portions of the variation for FHB or DON. However, specific markers could be identified as “top features” which may be associated with genes of interest which may operate closer to oligogenic vs. polygenic fashion (Supplementary Tables 2A,B). Biological endorsement of genomic features for FHB and DON were investigated through analysis of SNP effect annotations of markers on the 50 K SNP chip (Bayer et al., 2017). The top molecular markers with the highest mutual information are displayed in Supplementary Materials. Gene annotations generally concurred with resistance patterns.

**Figure 7:**
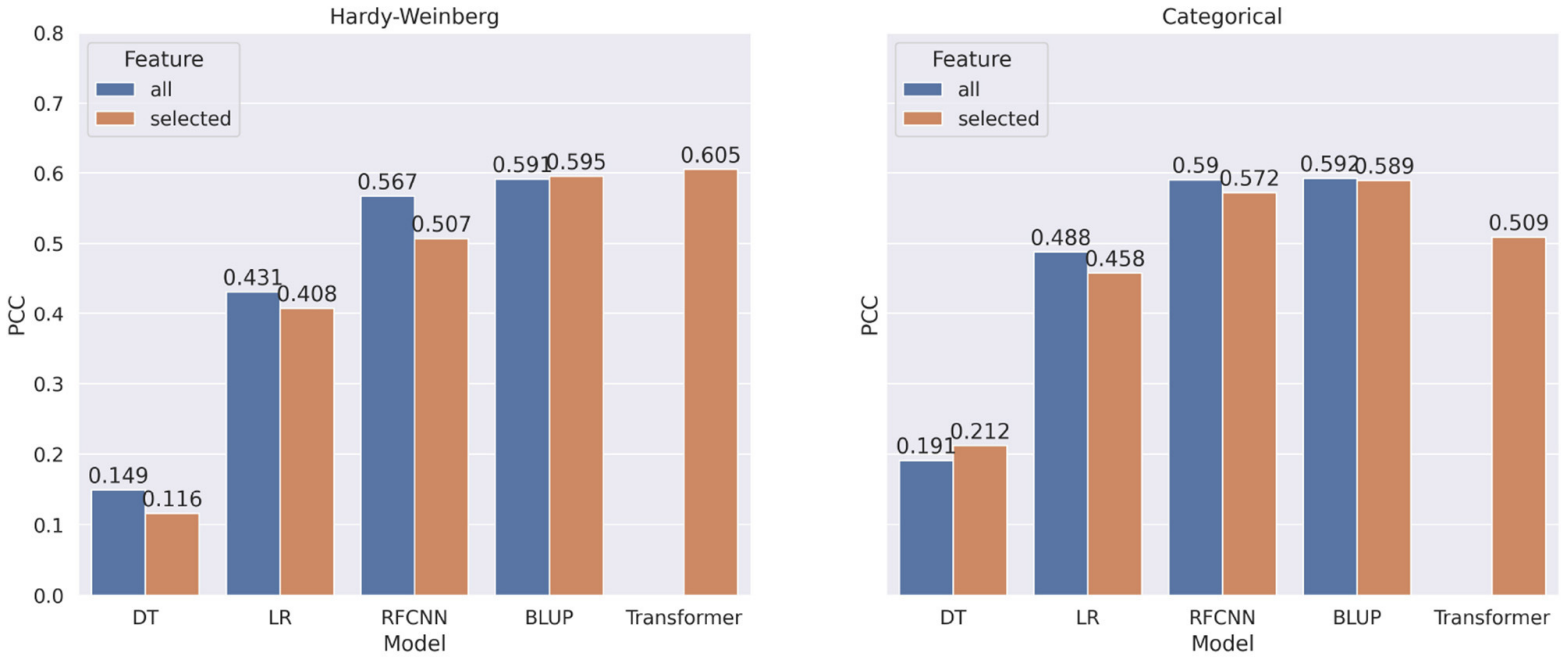
Comparison of pearson correlation coefficient when taking all markers as features vs. selected markers for DON. The PCC is measured between target and predicted DON. decision tree, linear regression, BLUP, Residual fully connected neural network and transformer are applied for each encoding technique.

In most of the experiments, when the machine learning or statistical methods are trained using all the markers, they performed better. Figure 7 shows the comparison of correlation score between models trained on all markers and models trained on selected markers for DON. Using marker frequency as features for the DON, only the BLUP method with reduced markers shows minor improvement (0.4%) over the BLUP method that uses all the markers. When the models were applied on categorical features for DON, only Decision Tree shows 2.1% improvement when reduced marker sets were used as features. Recall that due to memory issues, the Transformer model with the full set of markers was not executed, and thus is absent from Figure 7.

**Figure 8:**
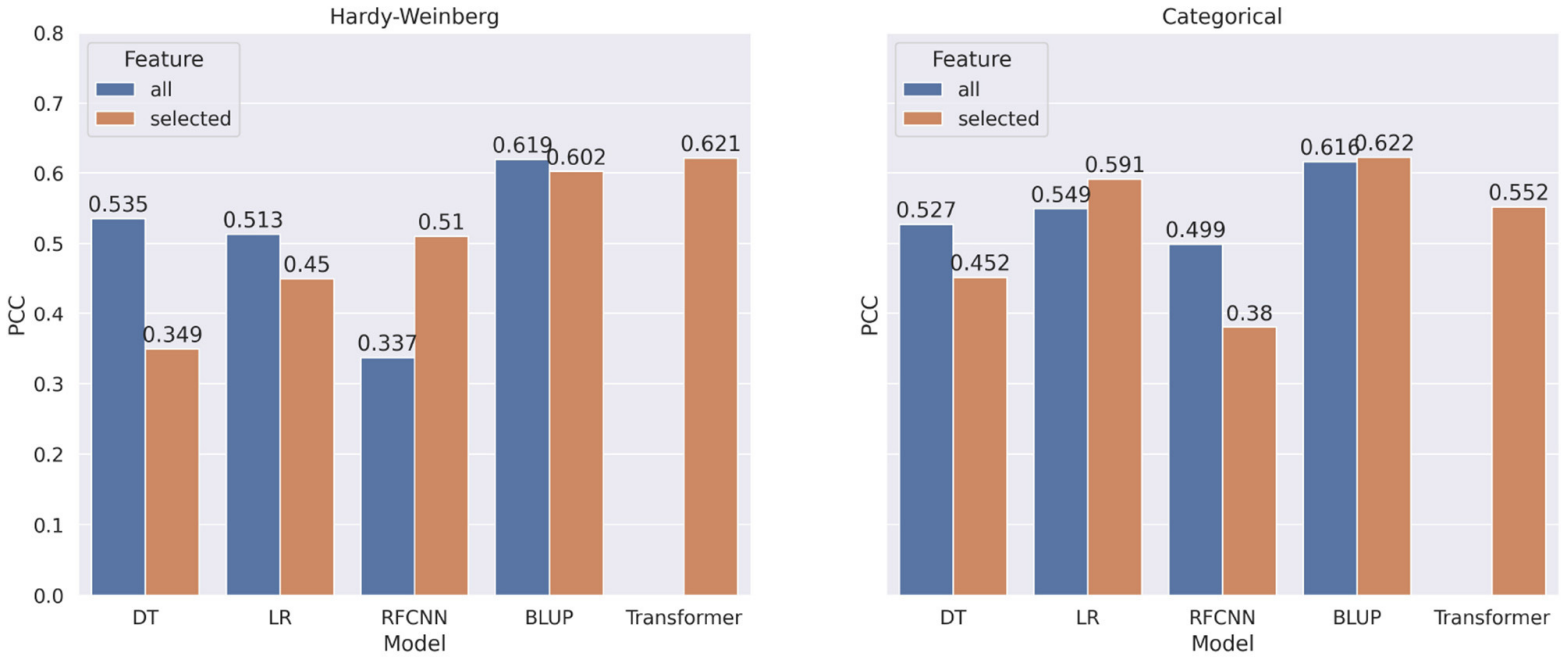
Comparison of pearson correlation coefficient when taking all markers vs. selected markers as features for FHB. The PCC is measured between target and predicted FHB.

In Figure 8, we show the comparison of correlation score between models trained on all markers and models trained on selected markers for FHB. From the figure, we observe a similar pattern for FHB that we observed for DON. In contrast to categorical encoding, when using marker frequency as a feature value, selected markers improve the performance of the FHB RFCNN model by 27.6%. For categorical encoding of features, Linear Regression with selected features shows 4.2% improvement overall features.

Though there is a significant increase in correlation score when all the markers are used for machine learning models, none of the BLUP models show any significant difference in performance when all markers or selected markers are used. Due to GPU memory limits, it was not possible to use all the features for Transformer architectures.

### 3.4. Best Performing Models

**Figure 9:**
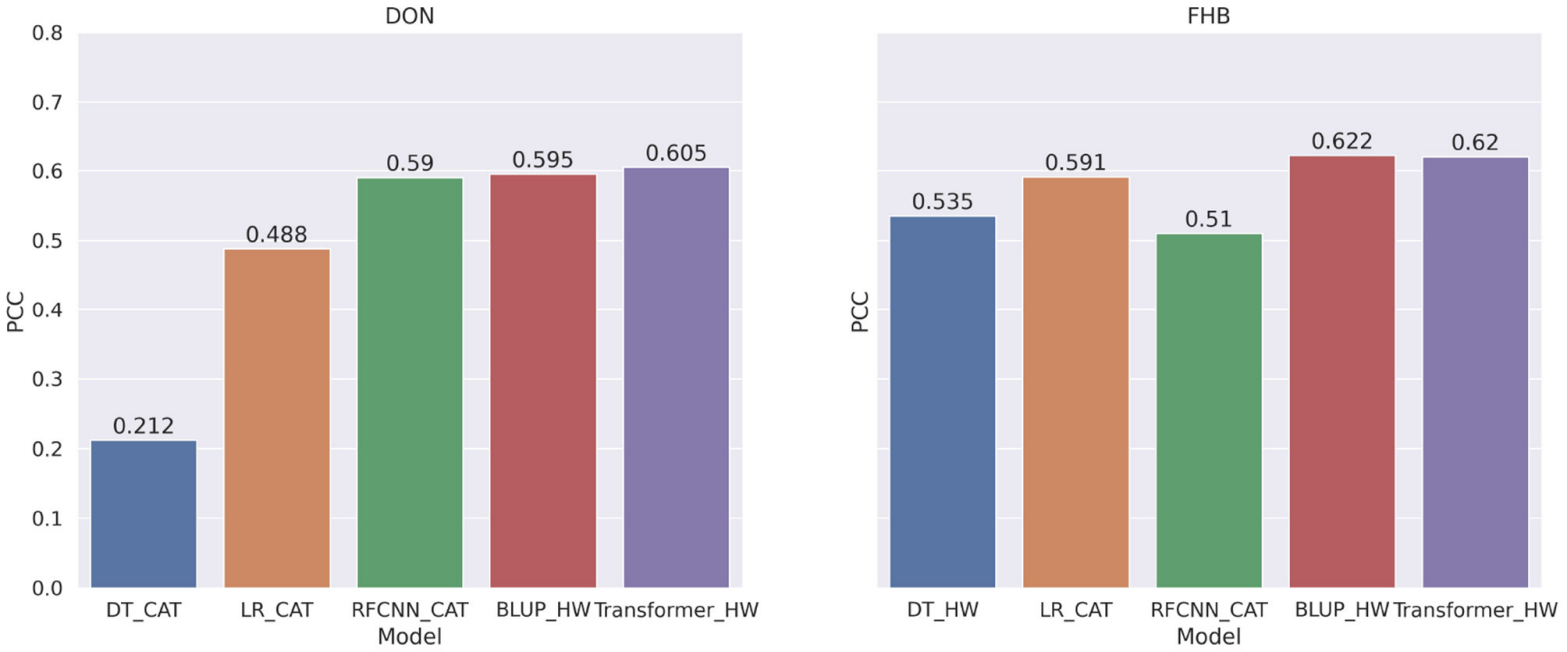
Comparison among the best models for each machine learning or statistical methods for DON and FHB. The PCC is measured between the target and predicted values of the phenotype.

Overall, BLUP and Transformer models that use genotype frequency as features obtained better correlation scores than other models. Figure 9 shows the comparison among the best models for FHB and DON. BLUP and Transformer's performance are competitive as we observe only 1% improvement over BLUP for DON and the same correlation score for FHB.

**Figure 10:**
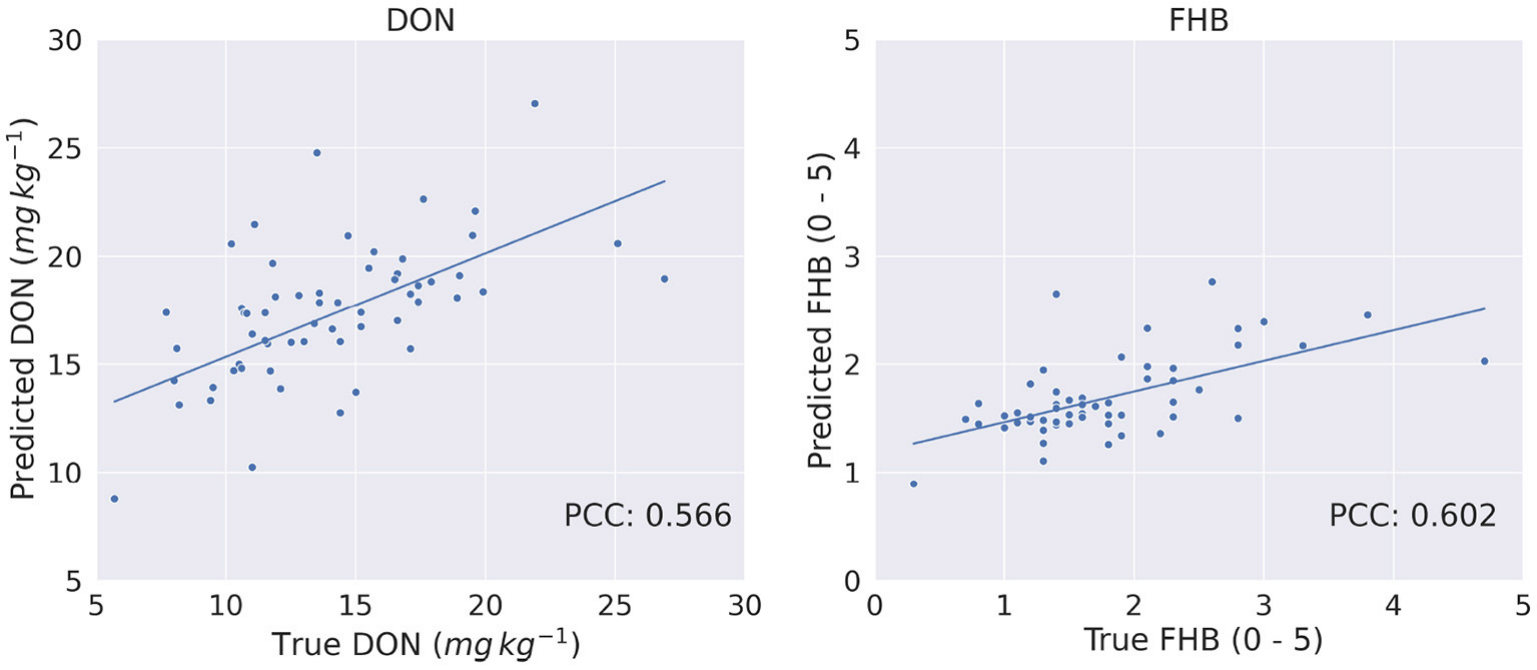
True vs. predicted phenotypes for DON (mg kg^−1^) and FHB (0 − 5) on the test set of 60 genotypes.

Figure 10 show the true vs. predicted phenotype score using Transformer. From the figures, we observed a linear relationship between the target and predicted score, which also shows that the Transformer architecture performs well to predict phenotypes.

### 3.5. Reliability of the GPTransformer Model

To understand how much reliable our GPTransformer model, we train and test the model in three separate traning-validation-test splits. With the GPTransformer model, the average PCC between the target and predicted phenotype for DON is 0.748 and for FHB is 0.703. The BLUP model obtained PCC of 0.789 and 0.681 for DON and FHB, respectively. The PCC we obtained with different splits using GPTransformer and BLUP models are not statisticallly significant as the *p*-value for DON is 0.576 and for FHB is 0.639.

## 4. Discussion

Transgressive segregation is common for FHB and DON in barley, where it is typical for offspring to deviate from mid-parent value. A greater degree of more susceptible genotypes was observed, which suggests that unique configuration of alleles over multiple loci may be responsible for resistance (Zhu et al., 1999). Such complexities in multiple resistance genes which form near-continuous distributions may not be accounted for under assumptions of statistical models examined herein. While the machine learning approach did not substantially surpass the statistical models in prediction, the ML approaches we have used here are capable of capturing non-additive genetic components. Thus, the predictions given may be incorporating some of these interactions in their prediction. However, we aren't able to tell what effects are being modeled in the ML models because of their opacity.

The current study confirms previous association mapping analysis of FHB and DON in barley (Massman et al., 2011), where quantitative trait loci (QTL) effects were small. While minor in nature, genes may additively contribute to resistance thereby lowering FHB and DON content. The advantage of such genes is that they do not typically carry issues seen when incorporating larger QTLs from exotics which tend to have tall stature or extreme heading date (Rudd et al., 2001). Under current study number of days to heading was not strongly associated with either character. Height was also weakly associated with DON content, however it did demonstrate moderate, negative relationship with FHB. Top feature molecular markers and genes identified for FHB and DON did not overlap, as one might predict based on their moderate-to-low trait correlation. Within barley, the relationship of FHB disease and DON content is not as robust as seen in other cereals such as wheat. Application of GEBVs based on DON content may offer a better target for developing resistance, since it is the primary factor monitored by industry.

Markers identified in the top features associated with FHB resistance were found on chromosomes 1H, 3H and 7H and all chromosomes for DON content, excluding 1H. Annotations of associated genes generally displayed direct biological function of resistance mechanisms. For instance FHB was associated with auxin transporter (HORVU1Hr1G073490) and response factor (HORVU7Hr1G033820), where this plant hormone has been associated with FHB severity and yield loss in barley (Petti et al., 2012). Also identified were genes involved in β-glucan synthesis cell (HORVU7Hr1G003460, HORVU7Hr1G003460), which may contribute to resistance via wall reinforcements or anti-oxidant properties (Martin et al., 2018). The molecular marker BOPA2-12-31203 in the top features group in this study previously identified by Huang et al. (2018) is a flanking marker for a QTL for FHB severity in the centromeric region of 7H. As a result of this toxic function, DON may induce programmed cell death (PCD, i.e., apoptosis). Development and Cell Death (DCD) domain protein (HORVU3Hr1G017930) and autophagy-related protein 18 (HORVU3Hr1G017150) underling removal of damaged cells may be involved in this process. Such top genomic features only explain a small percentage of total phenotypic variation for FHB and DON and could not be individually implemented under a marker-assisted selection program. However, biological functions associated with genes and markers highlighted above amongst others, may help explain why feature selections of a reduced marker subset may facilitate predictions with similar proficiency as when using all markers.

The proposed GPTransformer model takes the relationship among genetic markers into account within the model. The self-attention mechanism of the Transformer assigns a high weight to those markers that are associated with another specific marker. After applying the self-attention module, each obtained neuron is a combined representation of the genetic markers that are related to a specific marker. As many markers contribute toward a specific phenotype, GPTransformer has a unique attribute compared to other machine learning and statistical methods that takes marker relationships into account.

The frequency-based marker representation technique we applied for representing each allele carries more information as it indicates the zygosity and the frequency of the allele. The traditional categorical encoding (1, 0, and –1) only indicates the zygosity and remains the same for all the genetic markers. As each allele of a genetic marker is represented by its fixed frequency value, the frequency-based encoding provides us the information of the frequency as well as the zygosity. Though the frequency value remains the same within the same genetic marker for a specific allele, it may differ between different genetic markers. Thus, when the GPTransformer is combined with the frequency-based encoding, it performs better than the traditional categorical encoding-based model. The frequency-based representation is in the range of 0-1 and minimizes issues of vanishing gradient that may occur when training the GPTransformer or other neural network models.

The stability of the proposed model is tested with three different training and test data and the result shows the standard deviation of PCC on the test data for FHB is 0.04 and for DON is 0.09. The standard deviation of the BLUP model for FHB and DON for the same three different training and test is 0.008 and 0.04, respectively. The deviation from the average PCC for three different runs is higher for other machine learning methods we experimented with. This shows that the GPTransformer model is stable compared to other machine learning methods and as good as the popular BLUP model.

While the time commitment is higher and taking up to an hour to train the GPTransformer, it only took 24 epochs to complete training. The time complexity of the RFCNN is much lower than the Transformer though it took approximately 200 epochs to train. The most expensive task in the Transformer is the self-attention that requires substantial time and memory to complete. We ran both machine learning models on Intel Xeon E5-2690 v4 processor and NVIDIA Tesla P40 GPU, which contains 24 GB memory. With our Transformer architecture, we are able to fit all the markers that have mutual information ≥ 0.02. To fit all the genetic markers, a larger memory or multi-GPU instance is needed.

Though the performance of most of the machine learning methods improves when all the markers are used for prediction, the Transformer architecture outperforms other methods with selected markers. To the best of our knowledge, this is the first method that uses Transformer architecture for genomic prediction. This work showed that this method could outperform existing machine learning methods with fewer data and obtain state-of-the-art performance. Based on the performance in the language model domain, it is expected that with an increased amount of data, the performance of the Transformer model will also increase.

Our work shows the potential of the Transformer-based method for genomic prediction. Though Transformer generally performed well with a large amount of data in other fields, in this work, we showed that when trained on a small dataset, the Transformer encoder performs equally or better compared to the existing machine learning and statistical methods. As the genotype data generally contains many markers, calculating self-attention in a GPU will require a large amount of GPU memory that may not be available. Our feature selection step in the model addresses the memory issue of the Transformer method. This step reduces the number of markers and identifies the biologically relevant markers for a specific phenotype. We also applied genotype frequency-based encoding for each genotype. This encoding performs better when combined with the Transformer. If a large amount of data is available, the number of Transformer encoder blocks can be increased which may increase the overall performance.

2021 by Her Majesty the Queen in Right of Canada as represented by the Minister of Agriculture and Agri-Food Canada; This article is an open access article distributed under the terms and conditions of the Creative Commons Attribution (CC BY) license.

## Data Availability Statement

The datasets presented in this study can be found in online repositories. The names of the repository/repositories and accession number(s) can be found below: NCBI's Gene Expression Omnibus; GSE188791 (https://www.ncbi.nlm.nih.gov/geo/query/acc.cgi?acc=GSE188791).

## Author Contributions

SJ, JT, AB, WF, and MD: conceptualization and methodology. SJ, JT, and NH: software and data analysis. SJ and JT: writing—original draft preparation. AB, SJ, JT, MD, and NH: writing—review and editing. CH: microarray genotyping. AB, WF, and MD: supervision. AB, WF, and JT: project administration and funding acquisition. All authors have read and agreed to the published version of the manuscript.

## Funding

This research was funded by the Brewing and Malting Barley Research Institutes (BMBRI), Manitoba Crop Alliance, Growing Innovation: Agri-Food Research and Development Initiative (ARDI) project to WF and AB. Support was also delivered through part of the Barley Cluster project led by Alberta Barley with funding from the Western Grains Research Foundation and Agriculture & Agri-Food Canada (AAFC) under the Growing Forward 2 program.

## Conflict of Interest

The authors declare that the research was conducted in the absence of any commercial or financial relationships that could be construed as a potential conflict of interest.

## Publisher's Note

All claims expressed in this article are solely those of the authors and do not necessarily represent those of their affiliated organizations, or those of the publisher, the editors and the reviewers. Any product that may be evaluated in this article, or claim that may be made by its manufacturer, is not guaranteed or endorsed by the publisher.
